# Analysis of the Prognostic and Immunological Role of HSPB1 in Pituitary Adenoma: A Potential Target for Therapy

**DOI:** 10.3390/medicina59050885

**Published:** 2023-05-05

**Authors:** Sida Zhao, Bin Li, Yiyuan Chen, Chuzhong Li, Yazhuo Zhang

**Affiliations:** 1 Department of Cell and Biology, Beijing Neurosurgical Institute, Capital Medical University, No. 119, South Fourth Ring West Road, Fengtai District, Beijing 100070, Chinalibin@ccmu.edu.cn (B.L.);; 2 Neurosurgical Department, Beijing Tiantan Hospital, Capital Medical University, No. 119, South Fourth Ring West Road, Fengtai District, Beijing 100070, China

**Keywords:** HSPB1, pituitary adenoma, invasion, cavernous sinus, biomarker

## Abstract

*Background and Objectives*: The diagnosis and treatment of pituitary adenomas with cavernous sinus invasion pose significant challenges for clinicians. The objective of this study is to investigate the expression profile and prognostic value of HSPB1 (heat shock protein beta-1) in pituitary adenomas with invasive and non-invasive features. Additionally, we aim to explore the potential relationship between HSPB1 expression and immunological functions in pituitary adenoma. *Materials and Methods*: A total of 159 pituitary adenoma specimens (73 invasive tumours and 86 non-invasive tumours) underwent whole-transcriptome sequencing. Differentially expressed genes and pathways in invasive and non-invasive tumours were analysed. HSPB1 was subjected to adequate bioinformatics analysis using various databases such as TIMER, Xiantao and TISIDB. We investigated the correlation between HSPB1 expression and immune infiltration in cancers and predicted the target drug of HSPB1 using the TISIDB database. *Results*: HSPB1 expression was upregulated in invasive pituitary adenomas and affected immune cell infiltration. HSPB1 was significantly highly expressed in most tumours compared to normal tissues. High expression of HSPB1 was significantly associated with poorer overall survival. HSPB1 was involved in the regulation of the immune system in most cancers. The drugs DB11638, DB06094 and DB12695 could act as inhibitors of HSPB1. *Conclusions*: HSPB1 may serve as an important marker for invasive pituitary adenomas and promote tumour progression by modulating the immune system. Inhibitors of HSPB1 expression are currently available, making it a potential target for therapy in invasive pituitary adenoma.

## 1. Introduction

Pituitary adenomas are common intracranial tumours, with a rising incidence in recent years. While most pituitary adenomas are benign, a significant percentage exhibit invasive behaviour with the invasion of adjacent structures such as cavernous sinus (CS), sphenoid sinus and diaphragmatic sellae [1]. Cavernous sinus invasion is found in over 40% of aggressive pituitary adenomas [2]. The clinical diagnosis and treatment of pituitary adenomas with cavernous sinus invasion (CSI) are fraught with difficulties and challenges, such as difficulties in preoperative classification, inaccurate predictions of prognosis, incomplete tumour resections, postoperative recurrences and drug resistance [3]. Currently, there are no morphological features that distinguish invasive and metastatic lesions from non-invasive ones. Surgery is the first choice for most pituitary adenomas with CSI, but gross total resection is difficult and often results in residual tumour and high recurrence rates. There are no effective drugs for the treatment of pituitary adenomas with CSI except for lactotroph tumours. Therefore, exploring the effective biomarkers of pituitary adenomas with CSI will help to resolve the abovementioned difficulties in clinical diagnosis and treatment.

Heat shock protein beta-1 (HSPB1) belongs to the small heat shock protein (sHSP) family and is expressed in multiple cells and tissues. It is highly expressed in various types of tumours. HSPB1, also known as hsp27, is a multifunctional protein chaperone that plays roles in protein refolding and translation, cell differentiation and apoptosis, cytoskeletal maintenance and gene transcription [4,5]. Hsp27 can be upregulated by oxidative stress in cancers [6]. Several studies have confirmed that HSPB1 methylation is a biomarker in prostate cancer, breast cancer, colorectal cancer and malignant melanoma [7,8]. However, the expression of HSPB1 in pituitary tumours has not been reported.

Although there is increasing evidence that HSPB1 may play a vital role in the tumorigenesis of some specific types of cancers, a systematic analysis of HSPB1 in pituitary adenoma has not yet been conducted. Therefore, the aim of this study was to explore the expression profile and prognostic value of HSPB1 in pituitary adenoma and investigate the potential relationship between HSPB1 expression and immunological functions.

## 2. Materials and Methods

### 2.1. Patient SAMPLES

A total of 159 pituitary adenoma specimens were obtained from June 2018 to June 2019 in the Neurosurgery Oncology 3 Ward of Beijing Tiantan Hospital, Capital Medical University. This study was approved by the Beijing Tiantan Hospital Ethics Committee, and written informed consent was obtained from every participant (protocol code: KY 2021-032-02, date: 27 May 2021). Patients with pituitary adenoma were divided into two groups: the cavernous sinus non-invasion group (N-invasion) and the cavernous sinus invasion group (Invasion), according to the Knosp classification [9] based on preoperative MRI. The N-invasion group consisted of pituitary adenomas classified as Knosp grades 0–2, while the Invasion group comprised pituitary adenomas classified as Knosp grades 3–4. In addition, the criteria for classification also included intraoperative tumour invasion of the medial wall of the cavernous sinus. Patients with an intact medial wall of the cavernous sinus were placed in the non-invasive group, whereas those with a tumour-damaged medial wall were placed in the invasive group. The pituitary adenoma specimens were promptly frozen in liquid nitrogen within 30 min after surgery. All patients were followed up for 1 to 3 years after discharge.

### 2.2. RNA Sequencing and Data Analysis

The RNA-sequencing and data analysis were processed through the standard pipeline. Briefly, total RNA was isolated, and genomic DNA was removed using the AllPrep DNA/RNA Mini kit (Qiagen, Manchester, UK) according to the instructions. RNA Nano6000 assay kit (Aligent Technologies, Santa Clara, CA, USA) was used to assess the RNA concentration and quality. The sequencing library was generated by NEBNext^®^ UltraTM Directional RNA Library Prep Kit (NEB, Ipswich, MA, USA). The libraries were sequenced on an Illumina Hiseq X platform, and then 150 bp paired-end reads were generated. Clean reads were mapped to the human reference genome (NCBI37/hg19) using hisat2 (v2.0.5) to obtain read counts/FPKM/TPM for each noticed gene [10].

### 2.3. GO Functional Analysis and KEGG Pathway Analysis

GO functional analysis and KEGG pathway analysis were performed by https://www.bioinformatics.com.cn (last accessed on 7 January 2023), an online platform for data analysis and visualization [11].

### 2.4. HSPB1 Expression in Pan-Cancers

The mRNA expression of HSPB1 in different cancer types was analysed in the TIMER database (4 January 2023) (http://cistrome.org/TIMER/) [12] and Xiantao online tools (4 January 2023) (https://www.xiantao.love) [13]. The protein expression of HSPB1 in different cancer types was analysed in the Human Protein Atlas database (5 January 2023) (https://www.proteinatlas.org/) [14].

### 2.5. Survival Analysis in Pan-Cancers

The correlation between HSPB1 expression and survival in pan-cancers was analysed in the Xiantao online tools (6 January 2023) (https://www.xiantao.love) [13].

### 2.6. PPI Network Analysis

The interactional correlation of differentially expressed genes (DEGs) was assessed by the Search Tool for the Retrieval of Interacting Genes (STRING) online database (7 January 2023) (https://string-db.org) [15].

### 2.7. Differences Analysis in Immune Infiltration in Pituitary Adenomas

Differences analysis in immune infiltration in pituitary adenomas were performed in the TIMER database (5 January 2023) (http://cistrome.org/TIMER/) [12] according to RNA sequencing data.

### 2.8. Correlations between HSPB1 Expression and Immune System in Pan-Cancers

TISIDB database (6 January 2023) (http://cis.hku.hk/TISIDB) [16] was used to determine the relationship between HSPB1 expression and immune system.

### 2.9. Drug Prediction of HSPB1

Drug prediction of HSPB1 was performed in TISIDB database (9 January 2023) (http://cis.hku.hk/TISIDB) [16].

### 2.10. Statistical Analysis

SPSS25.0 statistical software package was applied for statistical analysis. All continuous variables were expressed as mean ± standard deviation (SD). The correlation of gene expression was evaluated using Spearman’s correlation. A *p*-value less than 0.05 was considered statistically significant.

## 3. Results

### 3.1. Basic Information of the Enrolled Cases

The study group consisted of 159 individuals, among whom 75 were females, and 84 were males. The study classified the tumours into two categories based on the Konsp grading criteria, which included 73 cases of invasive tumours and 86 cases of non-invasive tumours. Furthermore, the study group consisted of 37 individuals with recurrent pituitary adenomas and 122 individuals with primary pituitary adenomas. The basic clinical information of the enrolled individuals is presented in Table 1.

### 3.2. Analysis of Transcriptome Sequencing Results

**Figure 1:**
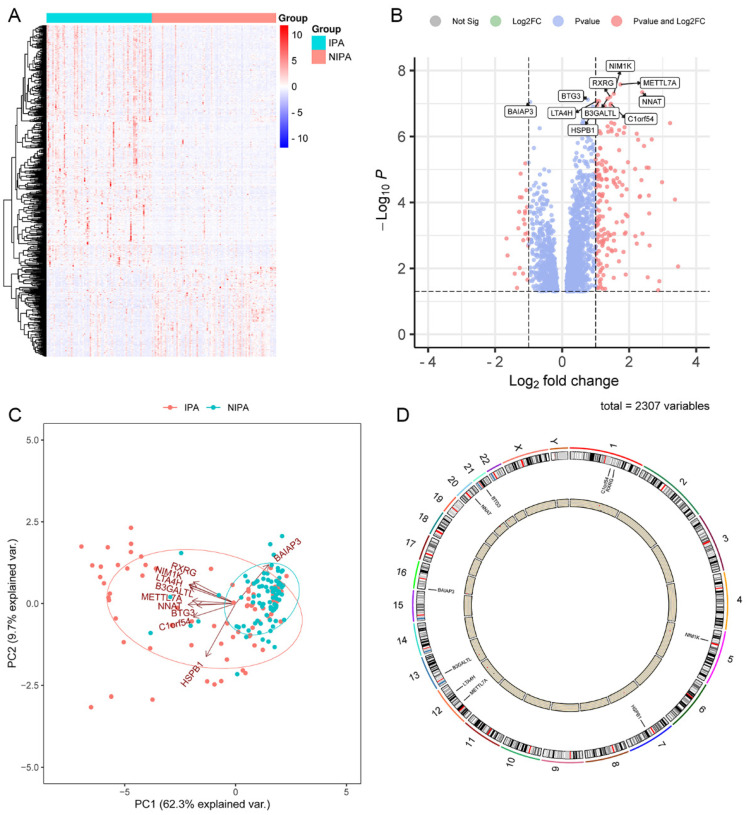
Gene expression differences between 73 invasive and 86 non-invasive tumours. (**A**) Heat map of gene expression differences. (**B**) Volcano map of gene expression differences. (**C**) Principal component analysis (PCA). (**D**) Differential gene chromosome loci.

Transcriptome sequencing was conducted on 73 invasive tumours and 86 non-invasive tumours. The results revealed differences in gene expression between the two groups of samples (Figure 1A). Using a difference multiple of 1.5 and a *p*-value of 0.05, the analysis identified 612 differential genes in the two groups, with the top 10 differential genes being C1orf54 (*p*-value = 1.01 × 10^−7^), BAIAP3 (*p*-value = 8.99 × 10^−8^), HSPB1 (*p*-value = 8.48 × 10^−8^), LTA4H (*p*-value = 8.41 × 10^−8^), BTG3 (*p*-value = 7.59 × 10^−8^), B3GALTL (*p*-value = 7.18 × 10^−8^), RXRG (*p*-value = 6.24 × 10^−8^), NIM1K (*p*-value = 5.02 × 10^−8^), NNAT (*p*-value = 4.56 × 10^−8^) and METTL7A (*p*-value = 2.63 × 10^−8^) (Figure 1B). These top 10 differential genes were able to categorize the 159 tumour samples into two groups (Figure 1C), demonstrating significant differences between invasive and non-invasive tumour samples under the influence of these genes. The chromosomal locations of these 10 differential genes are shown in Figure 1D.

### 3.3. GO Analysis and KEGG Enrichment Pathways of DEGs

**Figure 2:**
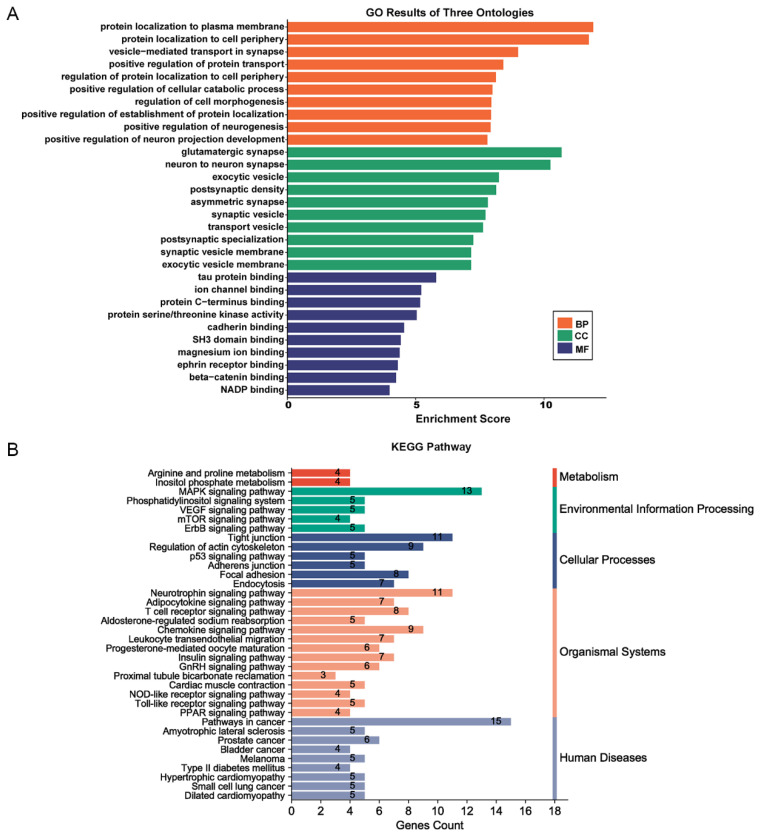
GO, KEGG pathway enrichment analysis. (**A**) GO function analysis. (**B**) KEGG pathway analysis.

The results of GO and KEGG pathway enrichment analysis showed that multiple signalling pathways associated with tumour development, such as protein transport, beta-catenin binding, cadherin binding, MAPK pathway, mTOR pathway, p53 pathway and VEGF pathway, were enriched by differential genes between invasive and non-invasive tumour groups (Figure 2).

### 3.4. PPI Network Establishment

**Figure 3:**
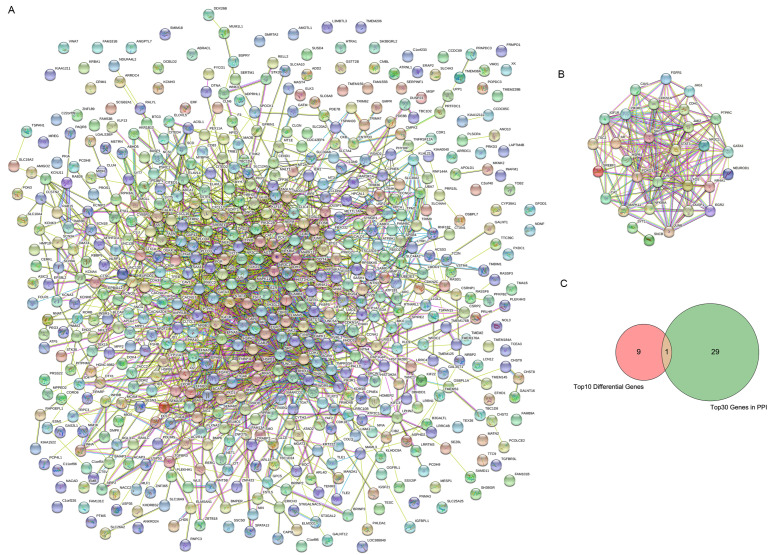
Protein interaction network. (**A**) Interaction network of the 612 differential genes. (**B**) Interaction network of the top 30 Degree-ranked genes. (**C**) Venn diagram of the top 10 differential genes versus the top 30 Degree-ranked genes.

The STRING website was used to analyse the interactions of 612 differential genes (Figure 3A). The Degree top 30 protein interactions network is shown in Figure 3B. The intersection of the top 10 differential genes with the top 30 proteins in the Degree ranking yields the HSPB1 gene (Figure 3C). It was demonstrated that the HSPB1 gene played an important role in the invasion of the cavernous sinus by pituitary adenomas and that the HSPB1 gene might be a key gene in the invasion of the cavernous sinus by pituitary adenomas. MAPK11, MAPK12, NFKBA, CAT, CAV1, FOS, CLU, RPS6KA1, NEFL, HSPA2, AR, G6PD, JUN, SRC, CALD1, HSPB8, STAT3, PRKCD and BAG3 are involved in the interactions with HSPB1.

### 3.5. Expression of HSPB1 and the Pathways Involved

**Figure 4:**
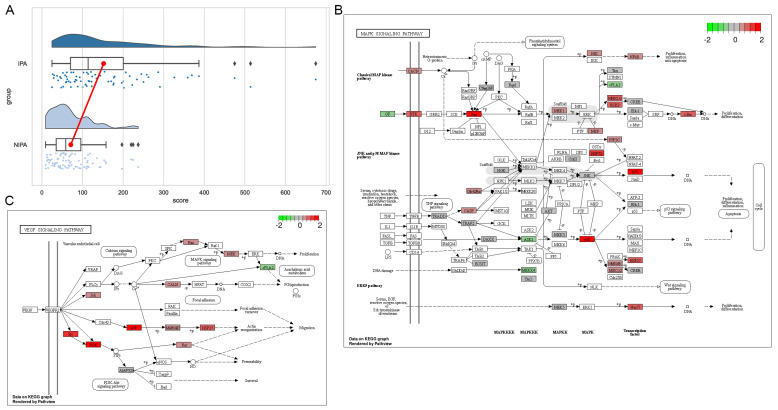
Expression of HSPB1 in tumours and the signalling pathways involved. (**A**) Expression of HSPB1 in invasive and non-invasive tumours. (**B**) Location of HSPB1 and other differential genes in the MAPK pathway. (**C**) Location of HSPB1 and other differential genes in the VEGF pathway.

The cloud and rain plots clearly demonstrated that the expression of HSPB1 was markedly upregulated in the invasive group compared to the non-invasive group, with a statistically significant difference (*p* < 0.0001) (Figure 4A). Furthermore, HSPB1 was found to be associated with various differential genes in the MAPK and VEGF pathways (Figure 4B,C). These results provide further evidence for the crucial role of the HSPB1 gene in facilitating the invasiveness of pituitary adenomas. The upregulation of the MAPK and VEGF pathways, which are known to play a critical role in tumour development, suggests that they may be involved in the progression of invasive pituitary adenomas as they invade the cavernous sinus.

### 3.6. Expression of HSPB1 in Pan-Cancers

**Figure 5:**
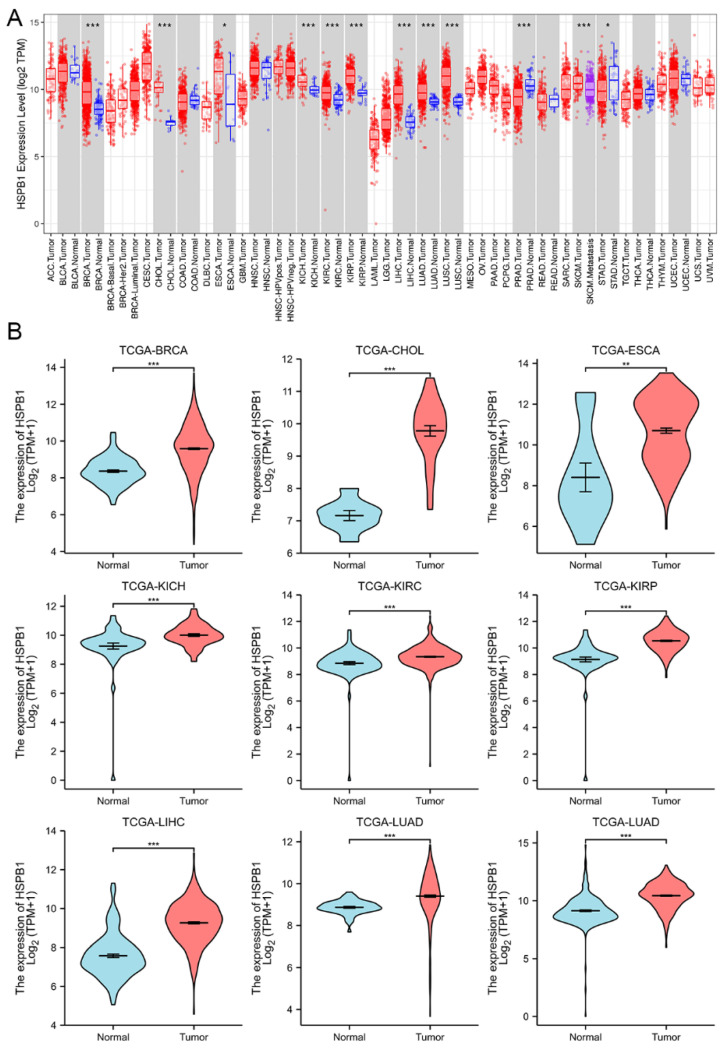
HSPB1 expression in pan-cancers. (**A**) Data from TIMER database. (**B**) Data from TCGA database. * *p* < 0.05; ** *p* < 0.01; *** *p* < 0.001.

**Figure 6:**
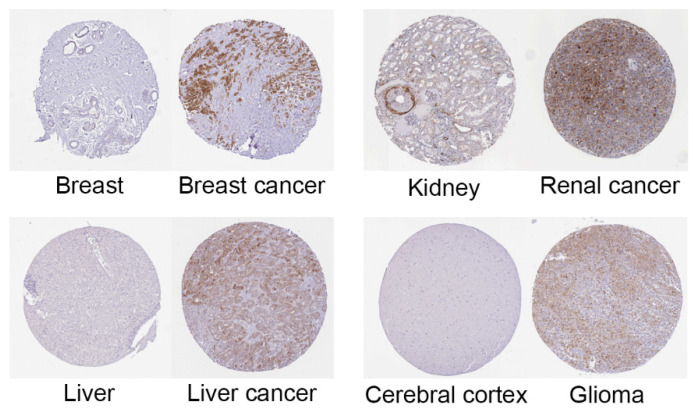
HSPB1 expression in the tumours of breast cancer, kidney cancer, liver cancer and glioma.

To understand the expression of HSPB1 in other tumours, we performed a pan-cancer analysis of HSPB1 using the TIMER database and the TCGA database (Figure 5). The results showed that data from both the TIMER database and TCGA database showed that HSPB1 was significantly highly expressed in most tumours compared to normal tissue. The expression of HSPB1 in the tumour tissues of breast invasive carcinoma (BRCA), cholangiocarcinoma (CHOL), oesophageal carcinoma (ESCA), kidney chromophobe (KICH), kidney renal clear cell carcinoma (KIRC), kidney renal papillary cell carcinoma (KIRP), liver hepatocellular carcinoma (LIHC), lung adenocarcinoma (LUAD) and lung squamous cell carcinoma (LUSC) was significantly higher than in the normal tissues (*p* < 0.05). The protein expression of HSPB1 in different cancer types was analysed in the Human Protein Atlas database. The results showed that the expression of HSPB1 in the tumour tissues of breast cancer, kidney cancer, liver cancer and glioma was higher than in the normal tissues (Figure 6).

### 3.7. Prognostic Analysis of HSPB1 in Pan-Cancers

**Figure 7:**
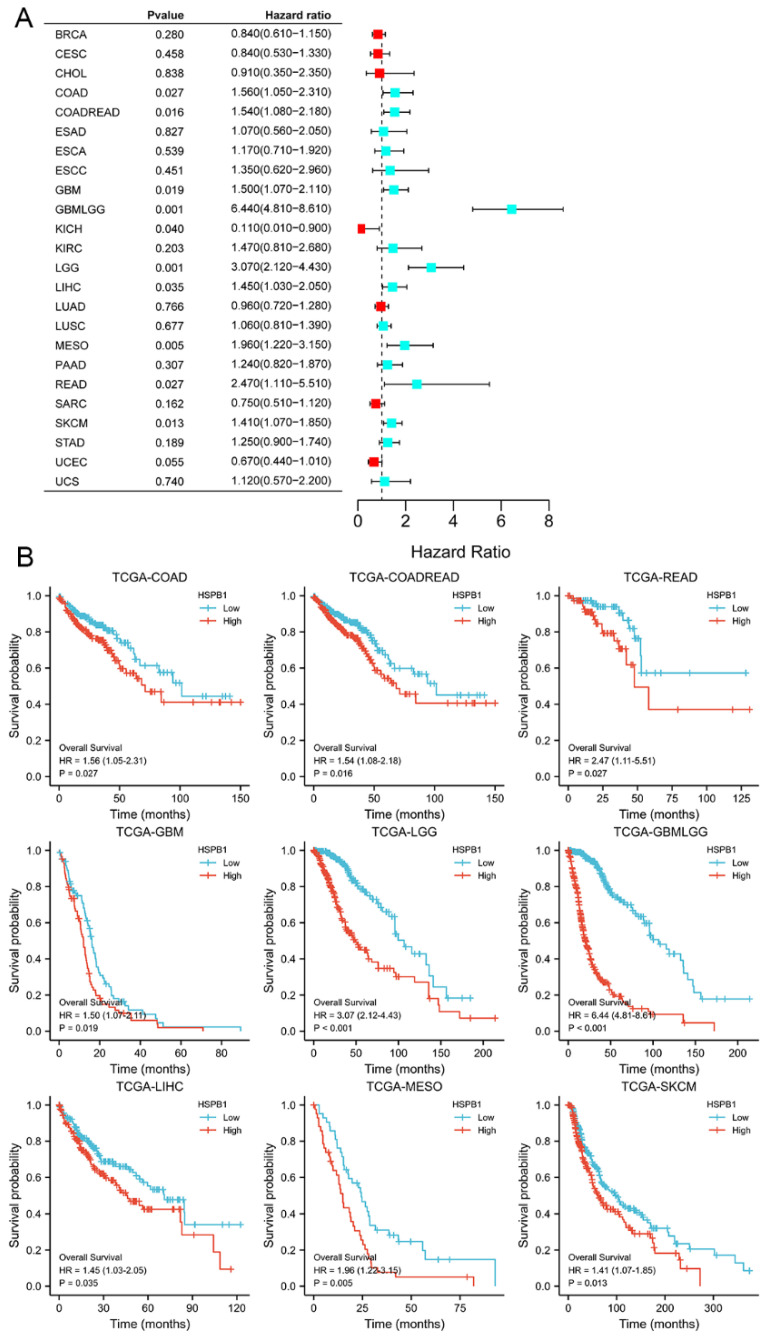
Relationship between HSPB1 and patients’ clinical prognosis. (**A**) Forest plot of HSPB1 in pan-cancer in relation to prognosis. (**B**) Survival curves for cancers with significant HSPB1 in relation to prognosis in the forest plot.

To evaluate the prognostic value of differential expression of HSPB1 in pan-cancers, the correlation between HSPB1 expression and survival data was determined. As presented in Figure 7, high expression of HSPB1 was significantly associated with poorer overall survival (OS) in colon adenocarcinoma (COAD) (*p* = 0.027), rectum adenocarcinoma (READ) (*p* = 0.027), glioblastoma multiforme (GBM) (*p* = 0.019), brain lower-grade glioma (LGG) (*p* < 0.001), liver hepatocellular carcinoma (LIHC) (*p* = 0.035), malignant mesothelioma (MESO) (*p* = 0.005) and skin cutaneous melanoma (SKCM) (*p* = 0.013).

### 3.8. Difference in Immune Cell Infiltration in Pituitary Adenomas

**Figure 8:**
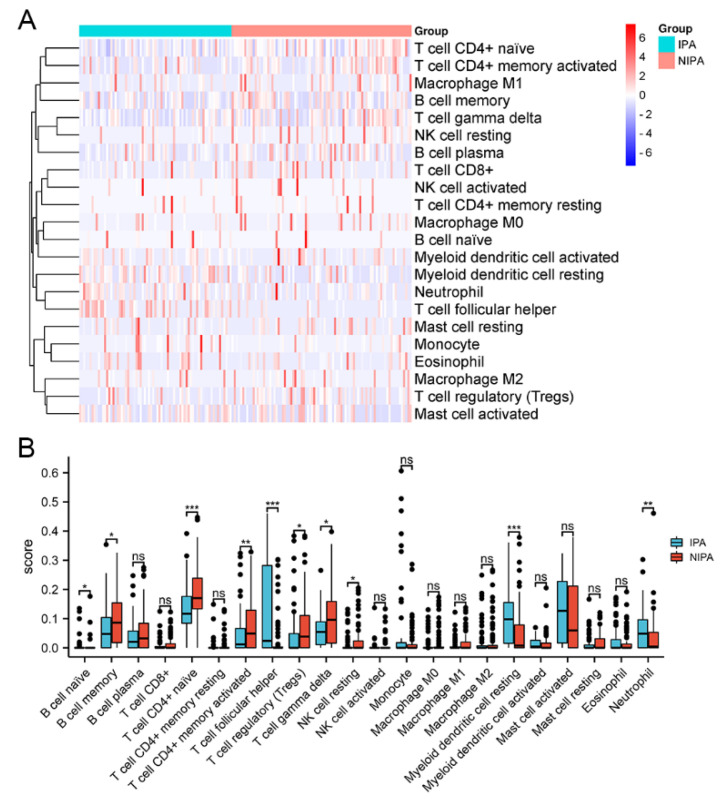
Differences in infiltration of 22 immune cell types between invasive and non-invasive tumour groups. (**A**) Heat map of differences in immune cell infiltration. (**B**) Box plot of differences in immune cell infiltration. * *p* < 0.05; ** *p* < 0.01; *** *p* < 0.001, ns: No statistical difference.

**Figure 9:**
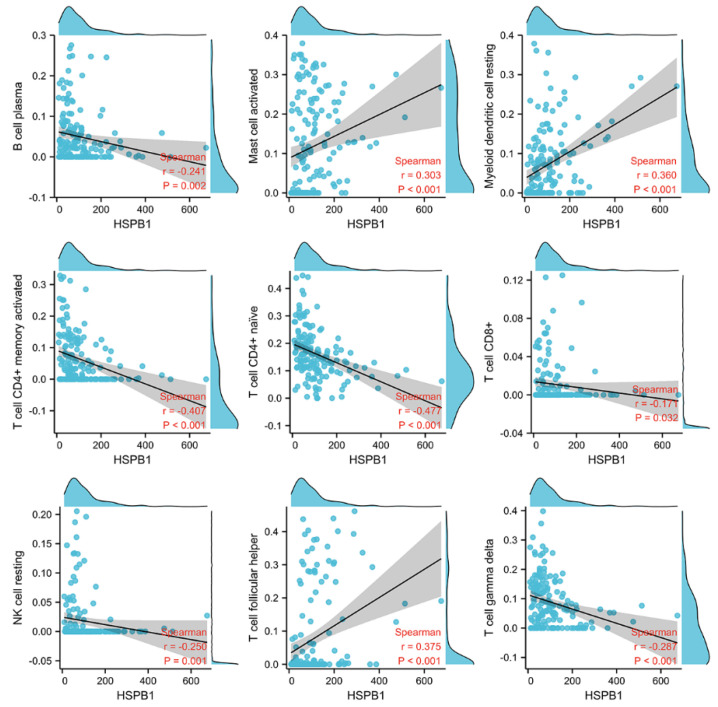
Correlation of HSPB1 expression with immune cell infiltration in 159 samples.

To investigate whether there is a difference in immune cell infiltration between invasive and non-invasive pituitary adenomas, we analysed 612 differential genes from the transcriptome data of 159 samples through the TIMER database. The results revealed a variety of immune cell infiltration differences between invasive and non-invasive tumours among the 22 immune cell types in the TIMER data (Figure 8). This suggests that the immune system may be involved in the development of invasive behaviour in pituitary adenomas. Further analysis revealed a correlation between HSPB1 expression and infiltration of a variety of immune cells (Figure 9). The high expression of HSPB1 was positively correlated with the infiltration of immune cells, such as mast cells, myeloid dendritic cells and T-cell follicular helper cells, and negatively correlated with the infiltration of immune cells such as plasma cells (*p* = 0.002), CD4-positive memory T cells (*p* < 0.001), CD4-positive initial T cells (*p* < 0.001), CD8-positive T cells (*p* = 0.032) and NK cells (*p* = 0.001). This indicates that in tumour tissue species, high expression of HSPB1 inhibits the exercise of functions by most immune cells.

### 3.9. Correlations of HSPB1 Expression and Immune Infiltration in Pan-Cancers

**Figure 10:**
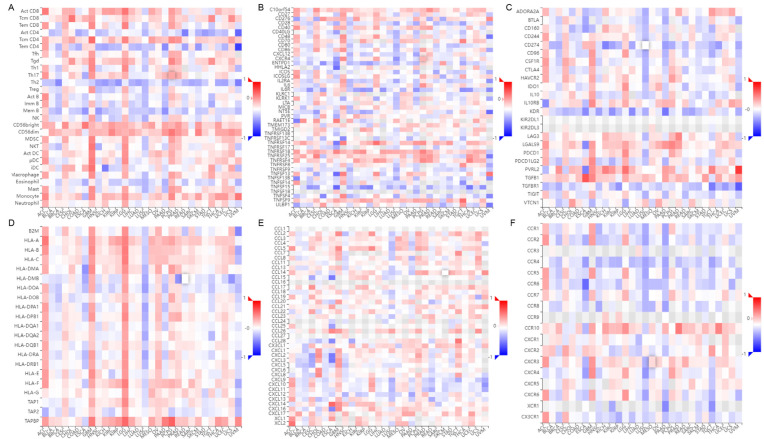
Correlation between HSPB1 expression and abundance of TILs (**A**), immune activators (**B**), immunosuppressants (**C**), major histocompatibility complexes (**D**), chemokines (**E**) and chemokine receptors (**F**).

To understand the correlation between HSPB1 expression and the immune system in pan-cancers, we conducted an analysis using the TISIDB website. The results showed that there was a correlation between HSPB1 expression and the abundance of tumour-infiltrating lymphocytes (TILs) (Figure 10A), immune activators (Figure 10B), immunosuppressants (Figure 10C), major histocompatibility complexes (Figure 10D), chemokines (Figure 10E) and chemokine receptors (Figure 10F) in pan-cancers. This suggests that in pan-cancers, HSPB1 may be involved in the regulation of the immune system.

### 3.10. Drug Prediction of HSPB1

**Figure 11:**
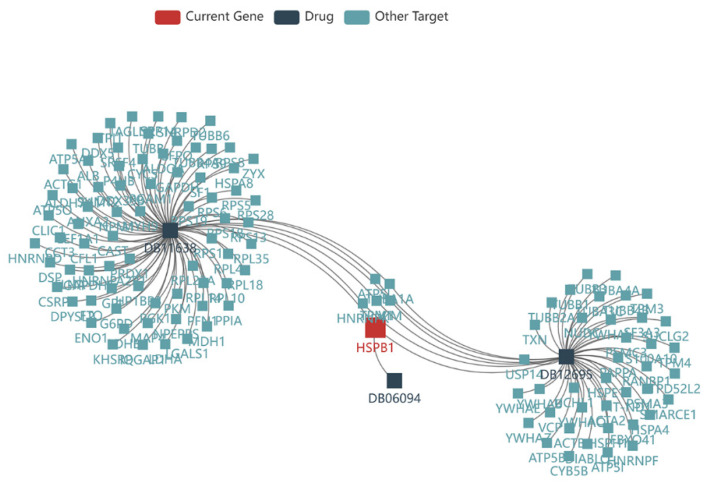
Drug prediction of HSPB1.

Drug prediction of HSPB1 was also performed in the TISIDB database. The results showed that the drugs DB11638, DB06094 and DB12695 could act as inhibitors of HSPB1 (Figure 11).

## 4. Discussion

Atypical morphological features, such as cavernous sinus invasion, indicate the aggressive nature of pituitary adenomas [17]. However, diagnosing and managing pituitary adenomas that invade the cavernous sinus can be challenging due to difficulty classifying them before surgery, inaccurate prognostic predictions, incomplete removal during surgery, and drug resistance [18,19]. While surgery is the primary treatment option for most pituitary adenomas that have invaded the cavernous sinus, complete removal can be difficult due to the potential risk of damaging the internal carotid artery and nerves that pass through the cavernous sinus. Additionally, residual tumours often lead to suboptimal endocrine remission and high recurrence rates. Besides prolactinomas, there are no effective drugs currently available for treating pituitary adenomas that invade the cavernous sinus [20].

The mechanisms by which pituitary adenomas invade the cavernous sinus are complex and not fully understood. Recent research has shown that there are molecular differences between invasive and non-invasive pituitary adenomas [21]. This information may help to identify the key biological features involved in the development of invasive pituitary adenomas and shed light on the mechanisms of invasion of the cavernous sinus. In our study, we screened for HSPB1 by analysing transcriptome sequencing data, a key gene in invasive pituitary adenomas, and performed adequate bioinformatics analysis. This will be useful for future basic research on invasive pituitary adenomas.

Heat shock proteins (HSPs) constitute a large family of molecular chaperones, classified by their molecular weight, which include HSP27, HSP40, HSP60, HSP70 and HSP90 [22]. HSPs are highly conserved molecular chaperones whose synthesis is induced by different stresses under environmental and pathophysiological conditions [23]. HSPs play a role in different physiological and protective processes, helping to maintain cellular homeostasis. In particular, they are involved in protein folding and maturation in response to various stresses [24]. Notably, HSPs also play a crucial role in various cancers, as they are associated with various cancer behaviours such as cell proliferation, metastasis and drug resistance [25].

HSPB1 is a multifunctional protein that regulates cell differentiation and development, and inhibits apoptosis in cancer cells [6]. Studies have reported that increased expression of HSPB1 is highly associated with poor prognosis in lung cancer through enhanced cell migration and invasion [26]. Our analysis of transcriptome sequencing results in 159 pituitary adenomas revealed that the HSPB1 gene was significantly highly expressed in invasive pituitary adenomas. Through bioinformatic analysis, we found that HSPB1 expression was upregulated in most cancers, such as breast cancer, kidney cancer, liver cancer and glioma. Additionally, high HSPB1 expression was associated with poor prognosis in these tumours. Our study also shows that HSPB1 plays an important role in multiple pathways, such as VEGF pathway and MAPK pathway. The aggressive behaviour of pituitary adenoma development involves activation of the MAPK pathway [27]. The activation of these pathways is closely linked to the development of tumours [28,29].

To investigate whether there is a difference in immune cell infiltration between invasive and non-invasive pituitary adenomas, we analysed 612 differential genes from the transcriptome data of 159 samples. The data showed that high expression of HSPB1 was negatively correlated with T-cell infiltration in 159 pituitary adenoma samples, which may predict an immunosuppressive function of HSPB1. However, little research has been reported on the association of HSPB1 with immunosuppression [30,31]. To understand the relationship between HSPB1 expression and the immune system in pan-cancers, we performed an analysis using the TISIDB website. The results showed that there was a correlation between HSPB1 expression and the abundance of TILs, immune activators, immunosuppressants, histocompatibility complexes, chemokines and chemokine receptors. This suggests that HSPB1 may be involved in the regulation of the immune system.

There are many publications on other potential treatment options in aggressive PAs, such as DNA repair interference with temozolomide (TMZ), epidermal growth factor-receptor (EGFR) inhibition, oestrogen receptor (ER) modulation, mammalian target of rapamycin (mTOR) inhibition, metalloprotease inhibition, peptide receptor radionuclide therapy (PRRT), radiotherapy, vascular endothelial growth factor (VEGF) inhibition and checkpoint inhibition. TMZ is an alkylating agent that interferes with DNA repair mechanisms and has been shown to have potential in treating aggressive PAs [32]. EGFR inhibition is another potential treatment option, as EGFR is often overexpressed in aggressive PAs [33]. ER modulation has also been explored as a potential treatment option, as some PAs are known to be hormone responsive [34]. mTOR inhibition has been shown to have potential in treating Pas with mutations in the mTOR pathway [35]. Metalloprotease inhibition has been explored as a potential treatment option, as metalloproteases are involved in the invasion and metastasis of Pas [33]. PRRT involves the use of radiolabelled peptides to target and destroy tumour cells [36]. Radiotherapy is also a potential treatment option for Pas, particularly those that are resistant to other forms of treatment [37]. VEGF inhibition has been explored as a potential treatment option, as VEGF is involved in the growth and spread of tumours [38]. Checkpoint inhibition involves the use of drugs that block immune checkpoints, allowing the immune system to better recognize and attack tumour cells [39]. Our study shows that the drugs DB11638, DB06094 and DB12695 could act as inhibitors of HSPB1 to treat invasive Pas. Overall, the treatment of Pas is complex and requires a multidisciplinary approach. Treatment options will depend on the specific characteristics of the tumour, as well as the patient’s overall health and medical history. Ongoing research is needed to develop new and more effective treatments for this challenging type of aggressive Pas.

## 5. Conclusions

In conclusion, our study showed that HSPB1 expression is upregulated in invasive pituitary adenomas and affects immune cell infiltration. Bioinformatic analysis showed that HSPB1 was highly expressed in pancytopenia and was associated with poor prognosis. HSPB1 may be involved in the regulation of the immune system in tumour initiation and progression. Drugs are currently available to inhibit HSPB1 expression. HSPB1 may be a potential target for the treatment of invasive pituitary adenoma. This study will be useful for future basic research into invasive pituitary adenoma.

## Figures and Tables

**Table 1:** Patient demographics.

Variable	Invasion (*n* = 73)	N-Invasion (*n* = 86)
Age (years)	48.2	49.7
Gender (no.)		
Male	27	57
Female	46	29
Lineage (no.)		
PIT-1	14	23
T-PIT	26	9
SF-1	19	48
Plurihormonal	8	4
Null cell	6	2
Recurrence (no.)		
Yes	28	9
No	45	77
Ki-67 (%)	5.2	4.2

## Data Availability

Publicly available datasets were analyzed in this study. These data can be obtained through the Materials and Methods section mentioned. The data of RNA Sequencing are available on request from the corresponding author.
